# Diversity of antigenic mutants of influenza A(H1N1)pdm09 virus escaped from human monoclonal antibodies

**DOI:** 10.1038/s41598-017-17986-8

**Published:** 2017-12-18

**Authors:** Atsuhiro Yasuhara, Seiya Yamayoshi, Priyanka Soni, Toru Takenaga, Chiharu Kawakami, Emi Takashita, Yuko Sakai-Tagawa, Ryuta Uraki, Mutsumi Ito, Kiyoko Iwatsuki-Horimoto, Tadahiro Sasaki, Kazuyoshi Ikuta, Shinya Yamada, Yoshihiro Kawaoka

**Affiliations:** 1 Division of Virology, Department of Microbiology and Immunology, Institute of Medical Science, University of Tokyo, Tokyo, Japan; 2 Yokohama City Institute of Public Health, Yokohama, Japan; 3 Influenza Virus Research Center, National Institute of Infectious Diseases, Shinjuku-ku, Japan; 4 Department of Virology, Research Institute for Microbial Diseases, Osaka University, Osaka, Japan; 5 Department of Pathobiological Sciences, School of Veterinary Medicine, University of Wisconsin-Madison, Madison, USA; 6 Department of Special Pathogens, International Research Center for Infectious Diseases, Institute of Medical Science, University of Tokyo, Tokyo, Japan; 7 ERATO Infection-Induced Host Responses Project, Japan Science and Technology Agency, Tokyo, Japan

## Abstract

Since the 2017 Southern Hemisphere influenza season, the A(H1N1)pdm09-like virus recommended for use in the vaccine was changed because human, but not ferret, sera distinguish A(H1N1)pdm09 viruses isolated after 2013 from the previously circulating strains. An amino acid substitution, lysine to glutamine, at position 166 (H3 numbering) in the major antigenic site of HA was reported to be responsible for the antigenic drift. Here, we obtained two anti-A(H1N1)pdm09 HA monoclonal antibodies that failed to neutralize viruses isolated after 2013 from a vaccinated volunteer. Escape mutations were identified at position 129, 165, or 166 in the major antigenic site of HA. Competitive growth of the escape mutant viruses with the wild-type virus revealed that some escape mutants possessing an amino acid substitution other than K166Q showed superior growth to that of the wild-type virus. These results suggest that in addition to the K166Q mutation that occurred in epidemic strains, other HA mutations can confer resistance to antibodies that recognize the K166 area, leading to emergence of epidemic strains with such mutations.

## Introduction

The first influenza pandemic of the 21st century began in 2009 with the emergence of the A(H1N1)pdm09 virus, which replaced the previous seasonal H1N1 (sH1N1) virus^1,2^. Surveillance of circulating A(H1N1)pdm09 viruses has revealed some genetic variations in the viral surface glycoproteins, hemagglutinin (HA) and neuraminidase (NA)^3,4^. However, until recently, the antigenicity of the circulating A(H1N1)pdm09 viruses was similar to the vaccine strain (A/California/7/2009) in assays with panels of antiserum obtained from infected ferrets^3^. Therefore, the World Health Organization (WHO) recommended using A/California/7/2009-like virus as a vaccine seed virus until the 2016–2017 Northern hemisphere influenza season^3^. However, human sera distinguished the antigenicity of recent A(H1N1)pdm09 viruses from that of the A/California/7/2009-like vaccine virus, whereas ferret antisera failed to detect this antigenic difference^5,6^. Accordingly, since the 2017 Southern Hemisphere influenza season, the WHO has recommended A/Michigan/45/2015-like virus be used as the vaccine seed virus^5,6^.

The HA protein is the major influenza viral antigen and the primary target of neutralizing antibodies^7^. A(H1N1)pdm09-HA has five major antigenic sites, which were identified by studies using A/Puerto Rico/8/34 (H1N1)^8–11^. Two immunodominant sites (Sa and Sb) are located proximal to the receptor-binding pocket and elicit high potency neutralizing antibodies^8,10^. The Ca sites (Ca1 and Ca2) are at the subunit interface, and the Cb site is close to the stalk region of HA.^8,10^. A(H1N1)pdm09 viruses isolated after the 2012–2013 influenza season, which are classified into the genetic group 6B, possess a lysine-to-glutamine substitution at position 166 (K166Q, H3 numbering) within the Sa antigenic site^12^. This mutation affects the antigenicity of recent A(H1N1)pdm09 viruses^13^. In the 2016–2017 influenza season, A(H1N1)pdm09 viruses classified into genetic group 6B.1 circulated among humans^5^. The 6B.1 viruses obtained a serine-to-asparagine substitution at position 165 (S165N) in the Sa antigenic site that resulted in the generation of an N-glycosylation site^4,14^.

Previous reports have described several human monoclonal antibodies that recognize an epitope around position 166 of A(H1N1)pdm09-HA and neutralize A(H1N1)pdm09 and sH1N1 viruses. However, these antibodies failed to neutralize A(H1N1)pdm09 viruses isolated after the 2012–2013 season, which possessed the K166Q mutation, or sH1N1 viruses isolated between 1986 and 2008, which had a potential glycosylation site (129-NHT-131) masking the epitope around position 166^15–17^. Middle-aged adults (i.e., born between 1965 and 1979) were reported to have a high antibody titer against the epitope around position 166 of the HA of A(H1N1)pdm09 viruses that were circulating before 2012, since they had been exposed to sH1N1 viruses that were circulating before 1985 and whose HA lacked the 129-NHT-131 glycosylation site. These middle-aged adults suffered from A(H1N)pdm09 virus infection with substantial morbidity and mortality during the 2013–2014 influenza season because of low neutralization antibody titers against the viruses possessing the K166Q substitution^12^. These reports demonstrate that the epitope around position 166 plays a role in the antigenic drift recently detected in assays with human, but not ferret, sera. Another group confirmed this phenomenon by using a panel of human monoclonal antibodies from a middle-aged adult^17^. *In vitro* selection of escape mutants from these monoclonal antibodies revealed that a mutation at position 166 of HA was responsible for the resistance to neutralization, suggesting that the antigenic drift was caused by the selective pressure of the human antibodies recognizing the epitope around position 166^17^.

Here, we established two human anti-A(H1N1)pdm09 HA monoclonal antibodies that recognized the epitope around position 166. We then attempted to obtain escape mutants possessing an amino acid substitution other than at position 166 and compared their growth ability with that of the wild-type virus.

## Results

### Establishment of human monoclonal antibodies recognizing A(H1N1)pdm09-HA

**Figure 1:**
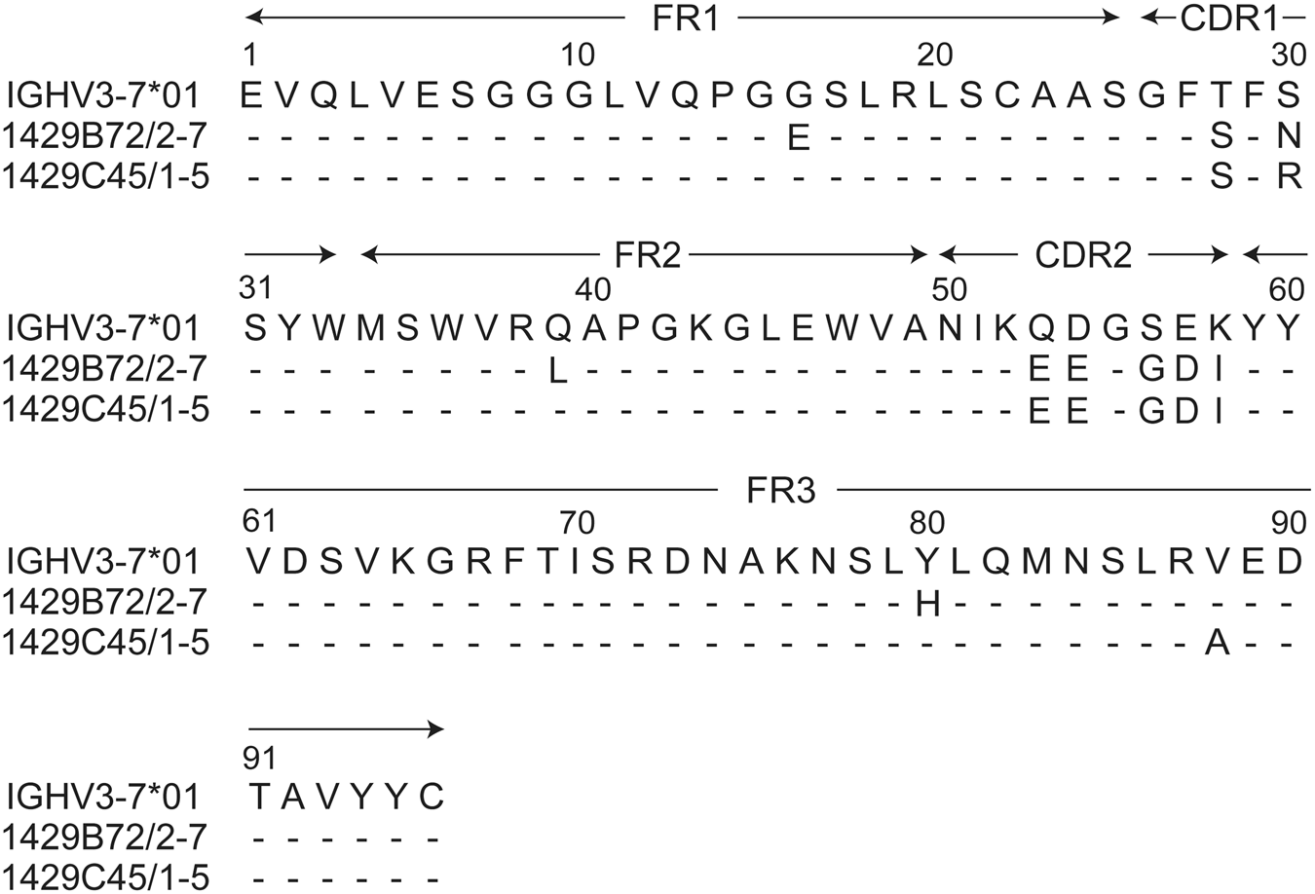
Sequence alignment of the variable regions of the heavy chains. The VH regions of 1429B72/2-7 and 1429C45/1-5 were compared with the germline sequence, IGHV3-7*01. Dashes indicate identities with respect to the germline sequence. The amino acid numbering corresponds to the Kabat numbering scheme.

PBMCs separated from a human volunteer vaccinated with the 2014–2015 seasonal influenza vaccine were fused with fusion partner SPYMEG cells to generate hybridomas that expressed a human antibody^18^. After screening by ELISA using recombinant H1-HA, hybridomas positive for anti-HA antibody production were biologically cloned. As a result of screening with 444 hybridoma clones, we obtained two monoclonal antibodies (mAbs), 1429B72/2–7 and 1429C45/1–5, which recognized A(H1N1)pdm09-HA but not H3, H5, H7, or type B-HAs. Nucleotide sequence analysis of the variable regions of each antibody (Table 1) revealed that the two monoclonal antibodies used the same VH and VL genes, suggesting that these two antibodies could be derived from the same B cell ancestor. The VH regions of 1429B72/2–7 and 1429C45/1–5 had 94.9% and 95.6% identities, respectively, compared with the germline sequence, IGHV3–7*01 (Fig. 1). Similar homologies were also observed for other genes (D, JH, VL, and JL). These results indicate that 1429B72/2–7 accumulated somatic mutations compared with 1429C45/1–5.

**Table 1:** Genetic features of human mAbs that recognize A(H1N1)pdm09-HA.

mAb	Heavy chain		Light chain	
	VH^a^	CDR3^b^	VL^c^	CDR3
1429B72/2-7	IGHV3-7*01	ARAGSYGDYVPYYNWFDS	IGKV3-15*01	QQYNNWPPWT
1429C45/1-5	IGHV3-7*01	ARAGSYGDYRPLYNWFDS	IGKV3-15*01	QQYNNWPPWT

^a^Variable genes for the heavy chain.

^b^Complementarity determining region 3.

^c^Variable genes for the light chain.

### Human mAbs fail to recognize A(H1N1)pdm09 viruses isolated after the 2013–2014 season

**Figure 2:**
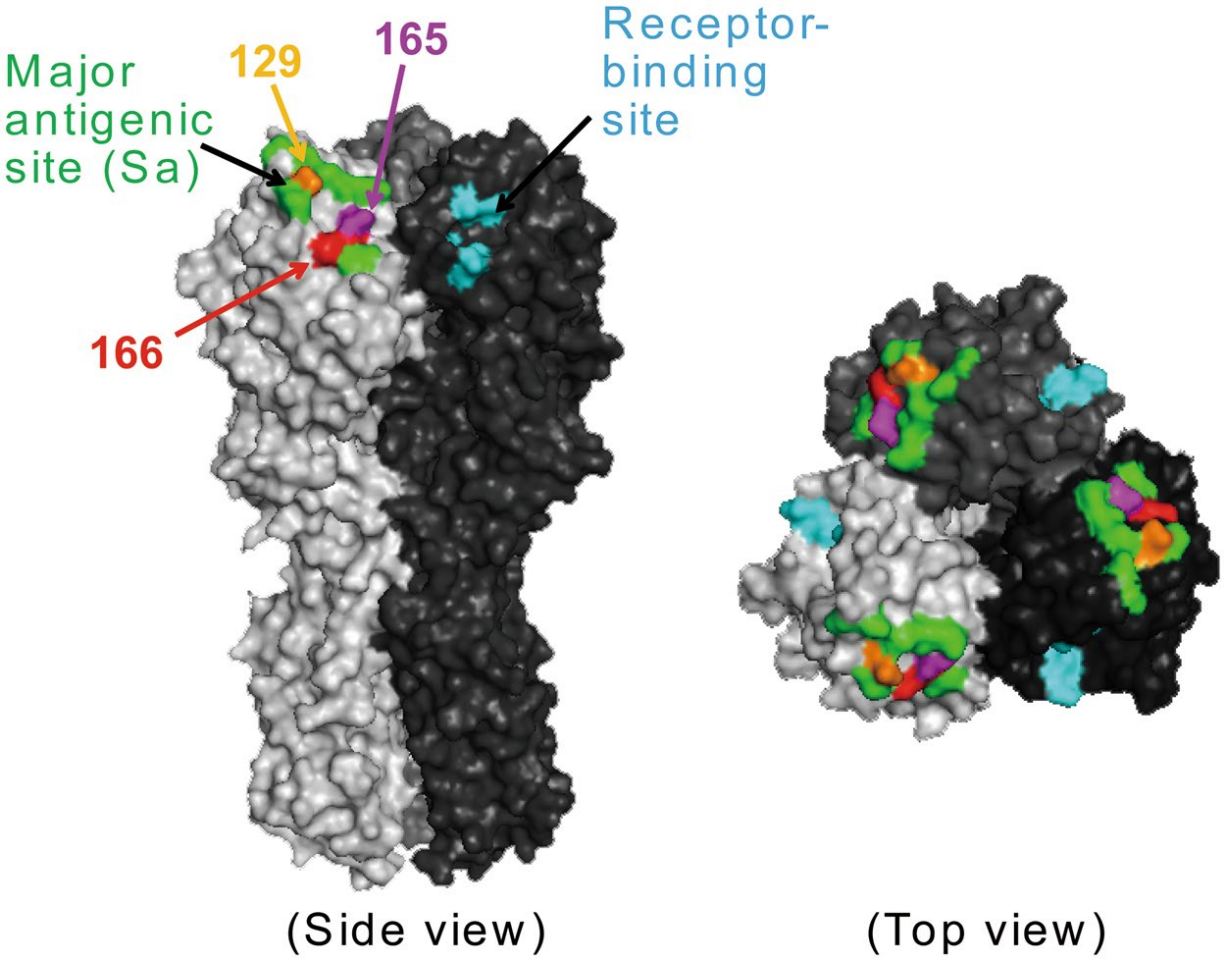
Positions 129, 165, and 166 on the HA molecule. Each HA monomer is indicated in white, gray, and black. Cyan indicates the residues involved in receptor binding; green indicates the Sa antigenic site. Amino acids at positions 129 (orange), 165 (magenta), and 166 (red) are shown on the HA of A/California/04/2009.

To characterize the two mAbs we obtained, we performed a microneutralization assay against A(H1N1)pdm09 viruses isolated in different influenza seasons. 1429B72/2-7 and 1429C45/1-5 neutralized A(H1N1)pdm09 viruses isolated between the 2009 and 2013 seasons with 50% inhibitory concentration (IC_50_) values of 0.08–0.12 and 0.24–0.39 μg/ml, respectively (Table 2). However, these mAbs failed to neutralize A(H1N1)pdm09 viruses isolated after the 2013–2014 season even at the highest concentration (50 μg/ml) tested. CR9114, which recognizes the HA stalk^19^, inhibited replication of all A(H1N1)pdm09 viruses tested, yielding an IC_50_ value of 0.92–9.92 μg/ml under the same experimental conditions. These results indicate that a key amino acid in the epitope recognized by the two mAbs changed after the 2013–2014 season. To identify this key amino acid substitution, we compared the amino acid sequences of A(H1N1)pdm09 HA obtained from the Global Initiative on Sharing All Influenza Data (GISAID). As previously reported^3,5^, this analysis revealed that A(H1N1)pdm09 viruses acquired the K166Q mutation in the major antigenic site Sa after 2013 (Table 3 and Fig. 2). The correlation between the loss of neutralization activity of the two mAbs and the acquisition of the K166Q mutation suggested that these mAbs may recognize an epitope that includes position 166.

**Table 2:** Neutralization activity of mAbs against A(H1N1)pdm09 viruses.

mAb	IC_50_ value (μg/ml) against A(H1N1)pdm09 virus isolated in						
	2009^a^	2010–2011^b^ (Clade 5^h^)	2011–2012^c^ (Clade 7)	2012–2013^d^ (Clade 6C)	2013–2014^e^ (Clade 6B)	2014–2015^f^ (Clade 6B)	2015–2016^g^ (Clade 6B.1)
1429B72/2-7	0.10	0.08	0.12	0.10	>50	>50	>50
1429C45/1-5	0.39	0.25	0.31	0.39	>50	>50	>50
CR9114	6.25	3.94	3.94	9.92	0.92	1.24	1.24

^a^A/California/04/2009, ^b^A/Hiroshima/66/2011, ^c^A/Osaka/83/2011, ^d^A/Osaka/33/2013, ^e^A/Osaka/6/2014, ^f^A/Yokohama/50/2015 and ^g^A/Yokohama/94/2015 were used in this experiment.

^h^Phylogenetic clades are listed for each isolate.

**Table 3:** Sequence variation of H1N1pdm09 HA at position 166.

Amino acid position^a^	Residue	Percentage of isolates possessing the indicated residue isolated in							
		2009–2010^b, d^	2010–2011^e^	2011–2012^f^	2012–2013^g^	2013–2014^h^	2014–2015^i^	2015–2016^j^	2016–2017^c, k^
166	K	99.1	96.4	97.7	72.4	4.6	0.5	0.3	0.5
	Q	0	0.1	0.4	7.7	93.8	98.2	98.9	99.1
	Others	0.9	3.5	1.9	19.9	1.6	1.3	0.8	0.4

^a^H3 numbering.

^b^Northern Hemisphere influenza season (from October to May).

^c^The sequences were obtained from October, 2016 to January, 2017.

^d^3616, ^e^1022, ^f^468, ^g^1057, ^h^1632, ^i^1139, ^j^5838, and ^k^424 HA amino acid sequences were used.

In previous reports^15–17^, some mAbs recognizing the epitope around position 166 of A(H1N1)pdm09 HA failed to neutralize sH1N1 viruses isolated between 1986 and 2008, because these viruses acquired an N-linked glycosylation at position 129, which likely covers the epitope that includes position 166. To investigate whether 1429B72/2-7 and 1429C45/1-5 have identical neutralization properties to previously reported 166-specific mAbs^15,16^, we tested the neutralization activities of our mAbs in a microneutralization assay using four sH1N1 viruses isolated in 1979, 1980, 1988, and 1992. As expected, our two monoclonal antibodies efficiently neutralized the sH1N1 viruses isolated in 1979 and 1980 but failed to neutralize the sH1N1 viruses isolated in 1988 and 1992, which contained the additional glycosylation site created by the K129N substitution, which shielded the epitope around position 166 (Table 4). CR9114 inhibited the replication of all of the sH1N1 viruses tested at an IC_50_ value of 12.50–17.68 μg/ml. These results indicate that our two mAbs recognized the epitope in a manner similar to that of previously reported mAbs^15–17^.

**Table 4:** Neutralization titers of mAbs against sH1N1 viruses.

mAb	IC_50_ value (μg/ml) against sH1N1virus isolated in			
	1979^a^	1980^b^	1988^c^	1992^d^
1429B72/2-7	0.28	0.28	>50	>50
1429C45/1-5	0.25	0.14	>50	>50
CR9114	17.68	12.50	15.75	12.50

^a^A/Kumamoto/37/79, ^b^A/Kamata/8/80, ^c^A/Tokyo/913/88, and ^d^A/Minato/131/92 were used in this experiment.

### Escape mutants from 1429B72/2-7 and 1429C45/1-5

Recent A(H1N1)pdm09 viruses possessing the K166Q mutation do not react with antibodies that recognize the epitope that includes position 166^5,12,17^. In addition, an *in vitro*-selected mutant possessing K166E did not react with such antibodies^17^. To examine whether antigenic variants possessing mutations at positions other than 166 would be selected by antibodies recognizing the epitope that includes position 166, we attempted to select escape mutant viruses in triplicate by passaging CA04 virus with various concentrations of 1429B72/2-7 or 1429C45/1-5. After 3–15 passages, we identified escape mutants for each mAb. These mutants possessed the N129D, S165C, S165N, K166E, or K166N mutation in their HA (Table 5). All of these mutations are located in the major antigenic site Sa (Fig. 2). No amino acid mutations were identified in the NA proteins. The results of selecting escape mutants indicate that an amino acid substitution at position 129 or 165, in addition to 166, conferred resistance to A(H1N1)pdm09 virus against human antibodies that recognized the epitope around position 166.

**Table 5:** Amino acid substitutions in the HA of escape mutants.

mAb	Amino acid substitution in the HA of the escape mutant		
	Clone 1^a^	Clone 2	Clone 3
1429B72/2-7	K166E^b^	S165C	N129D
1429C45/1-5	N129D	S165N	K166N

^a^Clones 1–3 were independently obtained.

^b^H3 numbering.

### Growth kinetics of the escape mutant viruses in cultured cells

**Figure 3:**
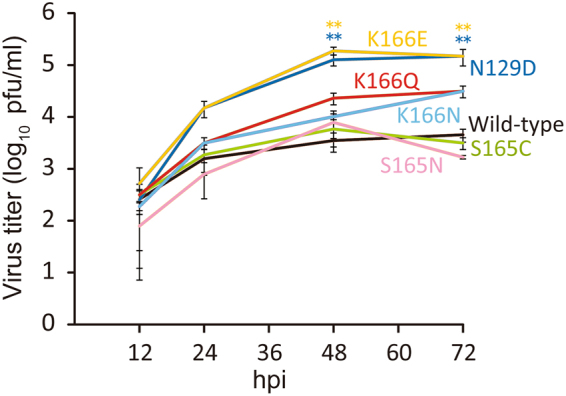
Replication kinetics of the wild-type virus and escape mutants. The growth kinetics of the wild-type CA04 virus and the indicated mutant viruses in A549 cells were compared. Cell culture supernatants of A549 cells infected at an MOI of 0.0001 were collected at 12, 24, 48, and 72 hpi. Virus titers are presented as the mean ± SD (n = 3). **P < 0.01 (two-way ANOVA followed by Dunnett’s tests).

To examine the fitness of the viruses possessing mutations that allowed them to escape from 1429B72/2-7 and 1429C45/1-5, we compared the growth kinetics of the escape mutant viruses in cultured cells with those of the wild-type virus. We infected A549 cells with the wild-type CA04 virus or a mutant virus possessing an escape mutation (i.e., HA-N129D, -S165C, -S165N, -K166E or -K166N) or the naturally occurring HA-K166Q substitution at a multiplicity of infection (MOI) of 0.0001. Virus titers at 12, 24, 48, and 72 hours post-infection (hpi) were determined by use of plaque assays with MDCK cells (Fig. 3). The virus with the N129D or K166E mutation replicated to higher titers than the wild-type virus (*P* < 0.01). The K166Q or K166N mutant viruses replicated to slightly higher titers compared with the wild-type virus. The S165C mutant virus replicated as efficiently as the wild-type virus, whereas the S165N mutant virus showed slightly lower titers at 12, 24, and 72 hpi. These results demonstrate that the fitness of some of the antigenic variant viruses in these human cells was increased by the escape mutation.

### Competitive growth of the escape mutant viruses with wild-type virus

**Figure 4:**
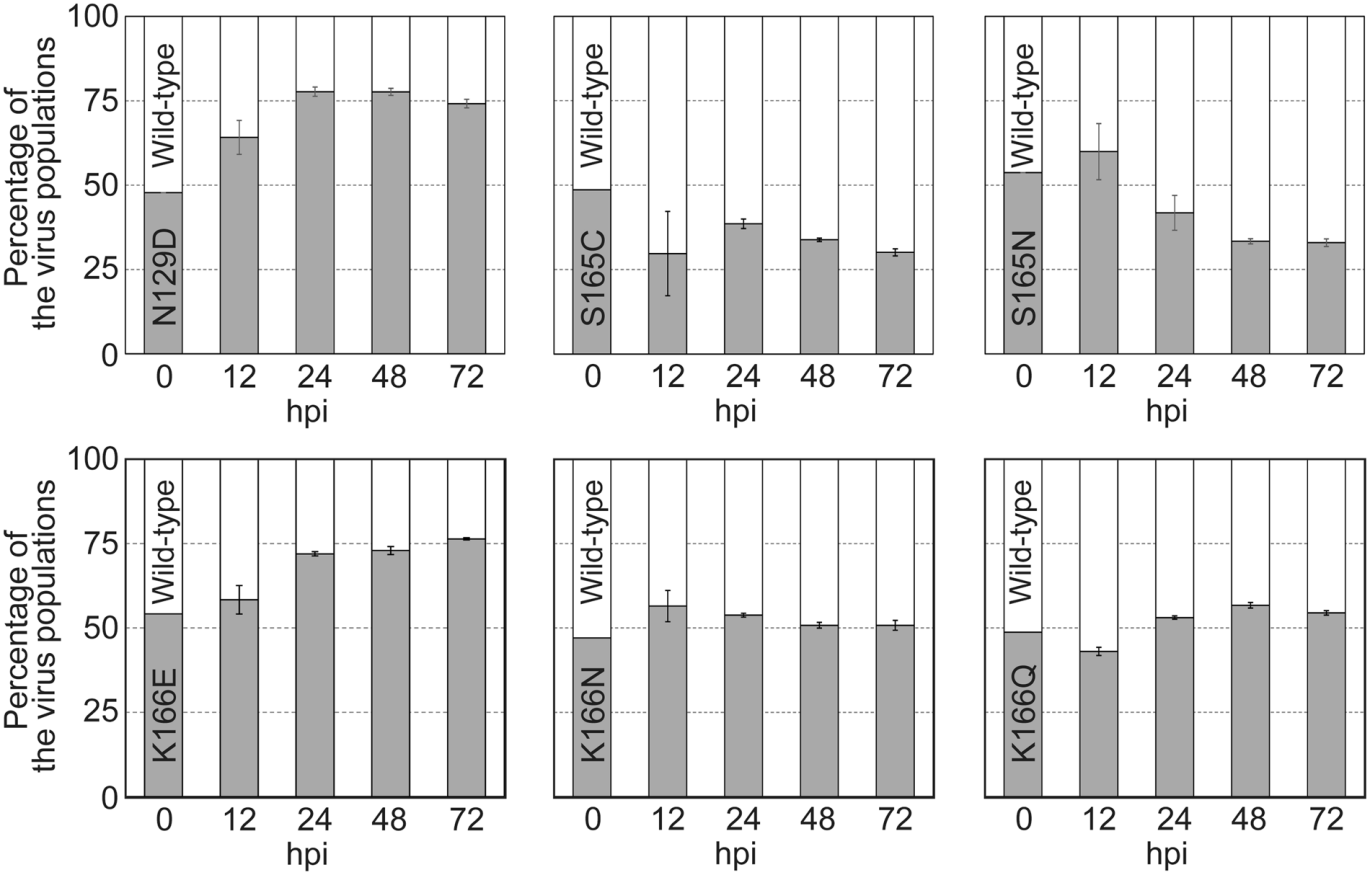
Competitive growth of escape mutant viruses with wild-type virus in cultured cells. The relative proportions of the indicated escape mutant virus and the wild-type virus are showed in the panels (grey bars show the percentage of mutant virus; white bars show the proportion of wild-type virus). The wild-type virus and the mutant viruses possessing each escape mutation were premixed at a 1:1 ratio based on their PFU titers. A549 cells were infected with the mixed viruses at an MOI of 0.0001, and supernatants were harvested at 12, 24, 48, and 72 hpi. The relative proportions of wild-type and mutant viruses were determined by using a QX200 droplet digital PCR system.

**Figure 5:**
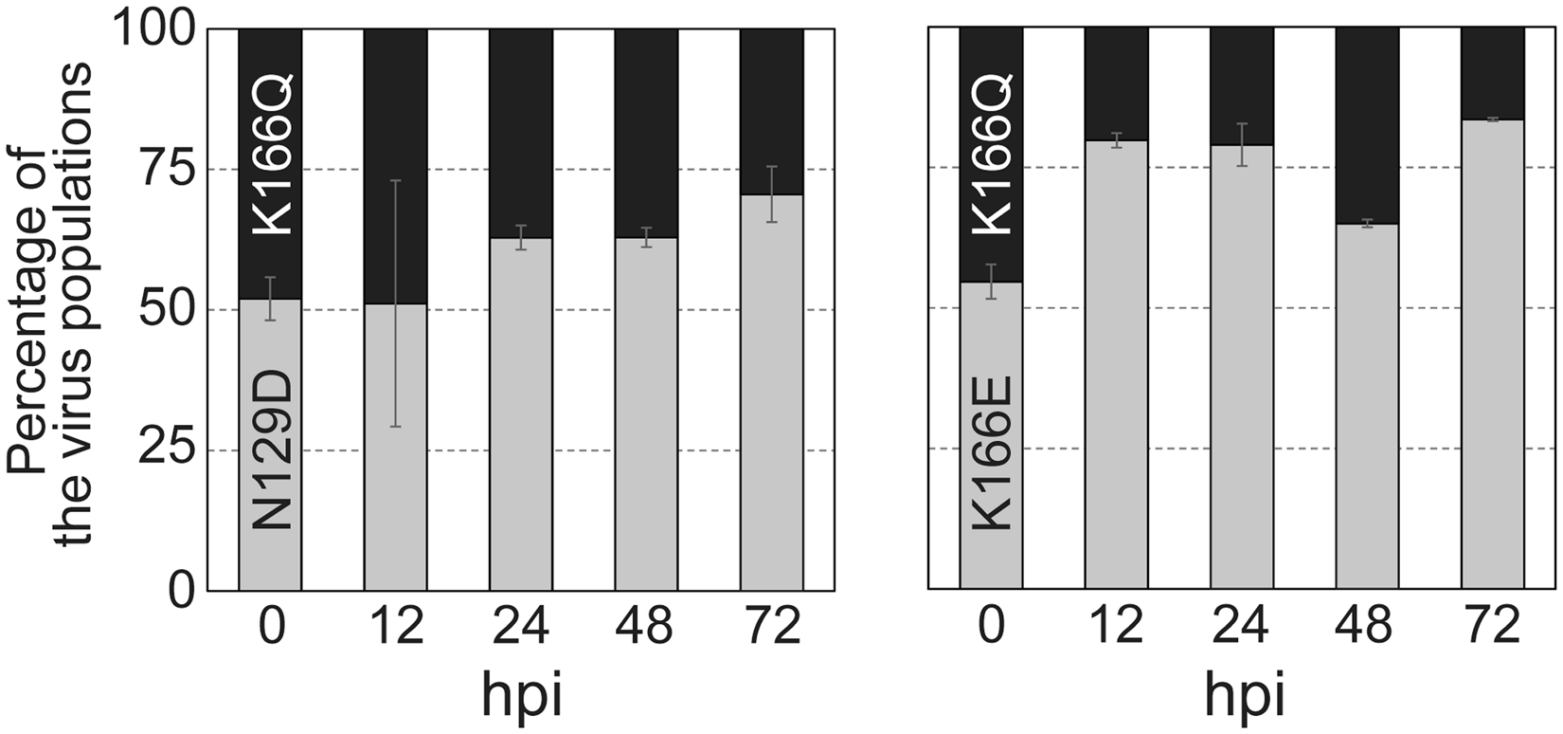
Competitive growth of escape variants with the naturally occurring K166Q mutant. The relative populations of the mutant viruses possessing HA-N129D or -K166E and the K166Q mutant virus were examined by using the competitive replication assay in A549 cells (MOI = 0.0001). The grey bars show the percentage of the mutant viruses with N129D or K166E and the black bars show the proportion of the K166Q mutant virus. The relative proportions of the indicated viruses at 12, 24, 48, and 72 hpi were determined by using ddPCR systems.

To further examine the fitness of the escape mutant viruses, we performed a competitive replication assay against the wild-type virus (Fig. 4). Briefly, we co-infected A549 cells with a 1:1 ratio of the wild-type CA04 virus and a mutant virus each possessing an escape mutation or the naturally occurring HA-K166Q. The populations of wild-type and mutant viruses were then measured by using a droplet digital PCR (ddPCR) system at 12, 24, 48, and 72 hpi. We found that the population of mutant viruses with HA-N129D or -K166E became dominant at 72 hpi (~75%), whereas the mutant viruses with HA-S165C or -S165N was detected at relatively low levels (~30%) at 72 hpi. The proportion of mutant viruses with HA-K166N or -K166Q remained at the same level as that of the wild-type virus at even 72 hpi. These results show that some escape mutant viruses are as competitive as the naturally occurring HA-K166Q mutant with the wild-type virus. Next, we performed the competitive replication assay using the HA-N129D or -K166E virus and the currently circulating HA-K166Q mutant virus (Fig. 5). Again, we co-infected A549 cells with a 1:1 ratio of the K166Q mutant virus and the N129D or K166E virus, and determined the populations of each mutant virus by using a ddPCR system at 12, 24, 48, and 72 hpi. We found that the relative proportion of mutant viruses with N129D or K166E became dominant over the K166Q mutant virus (70%‒84%), suggesting that the N129D or K166E mutant virus has the potential to overcome the currently circulating viruses as well as the pre-2013 isolates. Therefore, it remains unclear why the virus with HA-K166Q, rather than HA-N129D, -S165C, -S165N, -K166E, or -K166N became dominant in nature. Nevertheless, our results suggest that the escape mutants identified here had the potential to be dominant strains in the world.

## Discussion

Here, we established two human anti-A(H1N1)pdm09 HA monoclonal antibodies, 1429B72/2-7 and 1429C45/1-5, which failed to neutralize A(H1N1)pdm09 viruses isolated after 2013. By passaging the CA04 virus in the presence of these monoclonal antibodies, we obtained escape mutant viruses that possessed an amino acid substitution at position 129, 165, or 166. Some escape mutants showed better growth kinetics and became the dominant population in the competitive replication assay. These findings indicate that antigenic variant viruses with a mutation at position 129 or 165 could be selected by the immunological pressure of human antibodies that recognize the epitope around position 166 that are abundant in some individuals^12,17^, leading to the emergence of epidemic strains with such mutations.

Although an antigenic variant with the HA-K166Q mutation was selected in nature and became an epidemic strain in the 2012–2013 season, A(H1N1)pdm09 viruses possessing the N129D or S165N mutation temporarily appeared in the 2010–2011 season^20,21^. The S165N substitution did not affect the antigenicity of A(H1N1)pdm09 viruses in assays with ferret anti-serum^22,23^ but no information is available in this regard with human serum. The N129D substitution led to a significant reduction in the hemagglutination inhibition (HI) titer with human sera obtained from infected or vaccinated subjects^24^. Although viruses with the N129D or S165N mutation were obtained *in vitro* and those with N129D showed high replicative ability in the competitive replication assay, the viruses with N129D did not spread in human populations. These findings suggest that the emergence of antigenic drift strains is a stochastic event in which an epidemic strain emerges randomly from viruses with similar epidemic potential, or that factors other than HA antigenicity and replicative ability *in vitro* (e.g., transmissibility) play roles in determining which antigenic variants become epidemic strains. Nonetheless, the selection of antigenic variants *in vitro* provides useful information for identifying future epidemic strains. Further studies are needed to understand exactly what determines which antigenic mutants became dominant in the field.

1429B72/2-7 and 1429C45/1-5 did not neutralize A(H1N1)pdm09 isolates from after 2013 or sH1N1 isolates from after 1985 even at the highest concentration (50 μg/ml) tested. The neutralization properties of these two mAbs were similar to those of K166-specific antibodies that were reported previously^15–17^. In these previous studies, the K166-specific antibodies were found in individuals who were born before 1979, so they had been exposed to sH1N1 viruses that circulated prior to 1985 and lacked the glycosylation site at position 129^12^. 1429B72/2-7 and 1429C45/1-5 were obtained from a volunteer who was born in 1976, which is consistent with this pattern. The age in humans at which antigenic drift mutants are selected in nature is not known. However, our studies, together with those of others, suggest that at least the K166Q mutants may have been selected in individuals who had been exposed to sH1N1 that circulated prior to 1985, which lacked a glycosylation site near the 166 epitope^12,25^; namely, individuals who are over 30 years old.

Sequence analysis of variable heavy and light chain genes revealed that 1429B72/2-7 and 1429C45/1-5 share the VH3-7*01 gene, and somatic mutations are accumulated in 1429B72/2-7 compared with 1429C45/1-5. Together with the IC_50_ values against A(H1N1)pdm09 viruses, these data suggest that 1429B72/2-7 may have acquired its higher neutralizing activity via these accumulated somatic mutations. Nine human monoclonal antibodies, clones 4A10, 2O10, 4K8, 6D9, 2K11, SFV009-3F05, T2-9A, T2-6A, and T2-7D, which were reported to recognize the epitope around HA-166K of A(H1N1)pdm09 and to neutralize sH1N1 viruses isolated between 1986 and 2008, utilize the VH3-7*01 gene in combination with various DH, JH, VL, and JL genes^15–17^. Although the Sa antigenic site could be recognized by human antibodies harboring various kinds of VH genes^26,27^, the VH3-7*01 gene might play an important role in developing an antibody specific for the epitope that includes position 166 of both sH1N1-HA that circulated between 1986 and 2008 and H1N1pdm09-HAs that circulated before 2013.

In conclusion, amino acid substitutions other than at position 166 in major antigenic site Sa can be selected by the immunological pressure of antibodies, such as 1429B72/2-7 and 1429C45/1-5, that recognize an epitope including position 166. In combination with computational analyses and other methods^28^, *in vitro* selection of potential antigenic drift mutants may improve the selection of vaccine seed viruses.

## Methods

### Ethics

Human blood was collected from a volunteer by following a protocol approved by the Research Ethics Review Committee of the Institute of Medical Science, the University of Tokyo (approval number 25-58-1205), and all experiments in this manuscript were performed in accordance with the University of Tokyo’s guidelines and regulations. Written informed consent was obtained from all participants.

### Cells

Madin-Darby canine kidney (MDCK) cells were maintained in Eagle’s minimal essential medium (MEM) containing 5% newborn calf serum (NCS). A549 cells were maintained in Ham’s F-12K (Kaighn’s modification) medium containing 10% fetal calf serum (FCS). Human embryonic kidney 293T cells were maintained in Dulbecco’s modified Eagle’s medium (DMEM) containing 10% FCS. SPYMEG cells^18^ were maintained in DMEM containing 15% FCS. These cells were incubated at 37 °C under 5% CO_2_.

### Viruses

We used the following seven A(H1N1)pdm09 viruses: A/California/04/2009 (CA04), A/Hiroshima/66/2011 (clade 5), A/Osaka/83/2011 (clade 7), A/Osaka/33/2013 (clade 6C), A/Osaka/6/2014 (clade 6B), A/Yokohama/50/2015 (clade 6B), and A/Yokohama/94/2015 (clade 6B.1), and four seasonal H1N1 viruses: A/Kumamoto/37/79, A/Kamata/8/80, A/Tokyo/913/88, and A/Minato/131/92. All of these viruses were propagated in MDCK cells. Mutant CA04 viruses were generated by reverse genetics^29^ as described below.

### Plasmid-based reverse genetics

Mutant viruses used in the study were generated by plasmid-based reverse genetics, as described previously^29^. Briefly, plasmids encoding mutated CA04-HA were generated by a standard PCR technique based on pPol I encoding CA04-HA. The pPol I encoding CA04-HA and the remaining 7 pPol I plasmids were cotransfected into 293T cells along with eukaryotic protein expression plasmids for PB2, PB1, PA, and NP derived from A/Puerto Rico/8/34 by use of the TransIT 293 transfection reagent (Mirus) following the manufacturer’s instructions. At 48 h post-transfection, the supernatants containing viruses were harvested and inoculated into MDCK cells. All generated viruses were sequenced to ensure the absence of unwanted mutations. Primer sequences are available upon request.

### Hybridoma generation

Peripheral blood mononuclear cells (PBMCs) were isolated from a healthy volunteer, who was born in 1976 and vaccinated with the 2014–2015 seasonal influenza vaccine, by using Ficoll Paque Plus (GE Healthcare). To obtain hybridomas, the isolated PBMCs were fused with SPYMEG cells (MBL), which are fusion partner cells. The hybridomas were cultured in DMEM supplemented with 15% FCS in 96-well culture plates for 10–14 days in the presence of hypoxanthine-aminopterin-thymidine. The first screening for antibody characterization was performed by using an enzyme-linked immunosorbent assay (ELISA), as described below. Antibody-positive wells were cloned by several rounds of dilution.

### ELISA

96-well microtiter plates were coated with 2 μg/mL of recombinant HA derived from A/California/07/2009 (H1N1pdm09), A/Perth/16/2009 (H3N2), A/Egypt/N05056/2009 (H5N1), A/Netherland/219/2003 (H7N7), B/Florida/4/2006 (Yamagata-lineage), and B/Malaysia/2506/2004 (Victoria-lineage). All recombinant HAs were purchased from Sino Biological. After being blocked with 5 times-diluted Blocking One (Nakarai), these plates were incubated with the culture medium of the hybridomas. An HRP-conjugated goat anti-human IgG, Fcγ Fragment-specific antibody (Jackson Immuno-Research) was used as a secondary antibody.

### Nucleotide sequence determinations

Total RNA was extracted from hybridomas by using ISOGEN (Nippon gene). cDNA encoding the Fab region of a human antibody was PCR-amplified and directly sequenced. Determined nucleotide sequences of a heavy chain and a light chain were analyzed by using the IgBlast software (http://www.ncbi.nlm.nih.gov/igblast/).

### Purification of a human monoclonal IgG

Adaptation of hybridoma cells to serum-free medium, Hybridoma-SFM (GIBCO), was performed by passaging. The human antibody in the serum-free medium was purified by using a HiTrap rProtein A FF column (GE Healthcare) and the automated chromatography system ÄKTA pure 25 (GE Healthcare).

### *In vitro* microneutralization assay

To assess the neutralization capability of the antibodies, 100 TCID_50_ (Median tissue culture infectious dose) of each indicated virus in MEM containing 0.3% bovine serum albumin (BSA-MEM) were incubated with two-fold diluted antibodies (50–0.012 μg/mL) at 37 °C for 30 min. MDCK cells were washed with BSA-MEM and then incubated with the antibody-virus mixture in quadruplicate at 37 °C for 1 h. After the cells were washed twice with BSA-MEM, the cells were incubated with BSA-MEM containing 1 μg/ml L-(tosylamido-2-phenyl) ethyl chloromethyl ketone (TPCK)-treated trypsin for 3 days at 37 °C before the cytopathic effect (CPE) was examined. Antibody titers required to reduce virus replication by 50% (IC_50_) were calculated by using the Spearman & Kärber algorithm.

### Sequence analysis

Amino acid sequences of A(H1N1)pdm09 HA obtained from the Global Initiative on Sharing All Influenza Data (GISAID) were aligned. Percentages of viruses possessing the indicated amino acid at position 166 were calculated for each influenza season.

### Structural analysis

Amino acid positions were plotted on the crystal structure of CA04 HA (PDB accession code, 3LZG) using the PyMOL molecular graphics system to visualize the trimer.

### Selection of escape mutants

Escape mutants were selected by passaging CA04 virus in the presence of 1429B72/2-7 and 1429C45/1-5. Each two-fold diluted antibody was incubated with 10- or 100-fold diluted virus for 30 min at 37 °C. MDCK cells were washed and then incubated with the antibody-virus mixture at 37 °C for 1 h. After the inoculum was removed, the cells were incubated with BSA-MEM containing 1 μg/ml TPCK-treated trypsin for 3 days at 37 °C. Virus-containing supernatant was harvested from the CPE-positive well that contained the highest antibody concentration and was used for the next passage. We regarded a virus to be an escape mutant when it replicated well in the presence of mAbs at 50 μg/ml.

### Viral growth kinetics

Triplicate wells of confluent A549 cells were infected with the indicated viruses at a multiplicity of infection (MOI) of 0.0001, and incubated with Ham’s F-12K medium containing 0.3% BSA and 1 μg/ml TPCK-treated trypsin at 37 °C. Supernatants were harvested at 12, 24, 48, and 72 hours post-infection (hpi). Virus titers were determined by use of plaque assays with MDCK cells.

### Competitive replication assay

The mutant CA04 viruses possessing each indicated mutation and the wild-type virus or the K166Q mutant virus were premixed at 1:1 ratio based on their PFU (plaque forming unit) titers. Triplicate wells of confluent A549 cells were infected with the virus mixtures at an estimated MOI of 0.0001, and incubated with Ham’s F-12K medium containing 0.3% BSA and 1 μg/ml TPCK-treated trypsin at 37 °C. Supernatants were harvested at 12, 24, 48, and 72 hpi. The viral RNA was isolated from each supernatant using ISOGEN LS (Nippon gene). The relative proportions of wild-type and mutant viruses in each sample were determined by using a QX200 droplet digital PCR (ddPCR) system (Bio-Rad, Pleasanton, CA). FAM- and HEX-labeled primer/probe mixes were designed by the Bio-Rad online service. The ddPCR reaction mixture, consisting of ddPCR Supermix (Bio-Rad), the primer/probe mix, and the template cDNA, was loaded into a cartridge with droplet generation oil (Bio-Rad), and the cartridge was placed into the droplet generator (Bio-Rad). PCR amplification was carried out using the following thermal profile: 95 °C for 10 min, followed by 40 cycles of 94 °C for 30 s and 56 °C for 1 min, 1 cycle of 98 °C for 10 min, and ending at 4 °C. After amplification, the droplets from each well of the plate were read by the droplet reader (Bio-Rad) to count the number of positive and negative droplets. ddPCR data were analyzed with QuantaSoft analysis software (Bio-Rad), and the threshold was set manually at the highest point of the negative droplet cluster. The concentration of each target gene is determined by applying a Poisson distribution calculation across all droplets, and is presented as the number of copies per μl of PCR mixture. The percentage of virus populations shows the ratio of the copy number of mutant viruses and wild-type viruses.

### Statistical analysis

Statistical analysis (two-way analysis of variance (ANOVA) followed by Dunnett’s tests) was performed using GraphPad Prism software. *P* values < 0.01 were considered significantly different. No samples were excluded from the analysis.

### Data availability

All data analyzed during this study are included in this article.
